# Diagnostic reliability of magnetic resonance imaging for central nervous system syndromes in systemic lupus erythematosus: a prospective cohort study

**DOI:** 10.1186/1471-2474-11-13

**Published:** 2010-01-23

**Authors:** Yasuhiro Katsumata, Masayoshi Harigai, Yasushi Kawaguchi, Chikako Fukasawa, Makoto Soejima, Tokiko Kanno, Katsuji Nishimura, Takayuki Yamada, Hisashi Yamanaka, Masako Hara

**Affiliations:** 1 Institute of Rheumatology, Tokyo Women's Medical University, 10-22 Kawada-cho, Shinjuku-ku, Tokyo 162-0054, Japan; 2 Department of Pharmacovigilance, Graduate School, Tokyo Medical and Dental University, 1-5-45 Yushima, Bunkyo-ku, Tokyo 113-8519, Japan; 3 Department of Psychiatry, Tokyo Women's Medical University School of Medicine, 8-1 Kawada-cho, Shinjuku-ku, Tokyo 162-8666, Japan; 4 Department of Radiology, Tokyo Women's Medical University School of Medicine, 8-1 Kawada-cho, Shinjuku-ku, Tokyo 162-8666, Japan

## Abstract

**Background:**

Previous studies of magnetic resonance imaging (MRI) as a diagnostic tool for central nervous system (CNS) syndromes in systemic lupus erythematosus (SLE) contained several limitations such as study design, number of enrolled patients, and definition of CNS syndromes. We overcame these problems and statistically evaluated the diagnostic values of abnormal MRI signals and their chronological changes in CNS syndromes of SLE.

**Methods:**

We prospectively studied 191 patients with SLE, comparing those with (n = 57) and without (n = 134) CNS syndrome. CNS syndromes were characterized using the American College of Rheumatology case definitions.

**Results:**

Any abnormal MRI signals were more frequently observed in subjects in the CNS group (n = 25) than in the non-CNS group (n = 32) [relative risk (RR), 1.7; 95% confidence interval (CI), 1.1-2.7; *p *= 0.016] and the positive and negative predictive values for the diagnosis of CNS syndrome were 42% and 76%, respectively. Large abnormal MRI signals (ø ≥ 10 mm) were seen only in the CNS group (n = 7; RR, 3.7; CI, 2.9-4.7; *p *= 0.0002), whereas small abnormal MRI signals (ø < 10 mm) were seen in both groups with no statistical difference. Large signals always paralleled clinical outcome (*p *= 0.029), whereas small signals did not (*p *= 1.000).

**Conclusions:**

Abnormal MRI signals, which showed statistical associations with CNS syndrome, had insufficient diagnostic values. A large MRI signal was, however, useful as a diagnostic and surrogate marker for CNS syndrome of SLE, although it was less common.

## Background

Central nervous system (CNS) lupus is a serious and potentially life-threatening manifestation of systemic lupus erythematosus (SLE), occurring in 37-95% of cases, and is associated with an increased risk of death [1]. Despite its frequency and severity, the lack of a diagnostic gold standard makes it challenging to differentiate primary CNS lupus from secondary neuropsychiatric (NP) manifestations unrelated to SLE at their onsets [1-3]. The American College of Rheumatology (ACR) has developed a standardized nomenclature system that provides case definitions for 19 NP syndromes associated with SLE, including reporting standards and recommendations for laboratory and imaging tests [2]. Although this standardized nomenclature has helped to clarify a complicated situation, its usefulness as a clinical diagnostic criterion remains to be determined.

While neurological or psychological examinations are still the cornerstones for the diagnosis of CNS lupus, neuroimaging, electroencephalography and cerebrospinal fluid tests are also used [1,2,4-8]. Among conventional and more recent neuroimaging tools, conventional magnetic resonance imaging (MRI) still remains the modality of choice because of its availability and accessibility. Newer tools, such as magnetic resonance spectroscopy (MRS), magnetization transfer imaging (MTI), diffusion weighted imaging (DWI), perfusion weighted imaging (PWI), single photon emission computed tomography (SPECT), and positron emission tomography (PET) are still investigational (SPECT has probably been most established among these), although they have provided data to greatly improve our understanding of CNS lupus [4-7]. However, even the diagnostic validity and usefulness of conventional MRI is not yet fully established, because most published studies are small in size (n < 100, mostly < 50), retrospective, or based on non-standardized case definitions and some include only NPSLE, not SLE patients without CNS syndromes [2,4-7,9]. In order to overcome these problems of the previous studies we prospectively enrolled the largest number of SLE patients with or without active CNS syndrome which was categorized based on the ACR standardized case definitions and evaluated the accuracy and usefulness of conventional MRI in the diagnosis of CNS syndrome. We also assessed the suitability of conventional MRI as a surrogate marker for CNS lupus.

## Methods

### Patient population

This was a prospective study of 191 SLE patients with or without NP syndromes who were admitted to the Aoyama Hospital of The Tokyo Women's Medical University from August 1994 through October 2003. All these patients had 4 or more revised ACR (formerly, the American Rheumatism Association) criteria for SLE [10]. At our institution, those patients suspected of or newly diagnosed as having SLE were typically admitted for systemic evaluation regardless of the severity of the disease, and were eligible for inclusion in the study. Previously diagnosed SLE patients whose disease (NP syndrome or other manifestations of SLE) flared were also enrolled in the study. A total of 269 patients with SLE gave informed consent for inclusion in this study, including their MRI examinations during the above period. Among these 269 patients, those who had non-SLE-related NP manifestations arising from infection, uremia, electrolyte imbalance, hypoxia, brain tumor, trauma, primary mental disease or drug use (n = 45) or past histories of NP involvement (n = 33) were excluded prior to MRI scans. The patients were excluded because we wanted to compare recently (i.e. within a month) diagnosed active CNS lupus patients to non-NPSLE patients and because the unrelated conditions could affect current symptoms or laboratory and MRI findings. At the time of admission to the hospital, each patient completed a standardized medical history, including medication use, and had physical examinations that included neurologic and rheumatologic examinations. Psychiatric examinations were employed when indicated. Serology profiling for each patient was performed using standard immunoassays. The activity of SLE was measured using the SLE Activity Index (SLEDAI) [11]. Treatment with corticosteroids or immunosuppressive drugs was instituted as indicated following completion of these evaluations. One non-NPSLE patient at admission, who later developed NPSLE, was reevaluated at the onset of the NP syndrome (a mood disorder in this patient) and was reclassified into the CNS group. In this patient, data from the reevaluation, including the results of MRI, were used. Subjects were classified into the CNS group or the non-CNS group according to the presence or absence of active CNS syndromes. The CNS group was then further classified into the neurologic disorders group consisting of patients with neurologic disorders with or without other NP syndromes, or the psychiatric disorders group comprising patients with psychiatric disorders with or without other NP syndromes [2,4,8,12]. Thirteen patients were classified into both neurologic and psychiatric disorders groups. Detailed diagnostic criteria for these groups are described below. The study was approved by the Ethical Committee of our institution and the Helsinki declaration was followed throughout the study.

### Diagnosis of CNS lupus

Although ACR nomenclature and case definitions include 12 CNS syndromes and 7 peripheral nervous system syndromes [2,4,8,12], we included only the 12 CNS syndromes in the present study because of the substantial differences in anatomy, function, and clinical characteristics between the central and peripheral nervous systems. Slight or mild cognitive dysfunction without significant clinical impairment, as revealed by the detailed neuropsychological tests described below in "*Evaluation of psychological impairment*," was excluded from the CNS syndromes in our study. Using published normative data, patients in this category (n = 3) exhibited less than the median of normal controls by 2 standard deviation (SD) in 2 or more of areas of cognitive function without significant clinical impairment [13]. Tension headache, or episodic tension type headache, was also excluded from our study.

The final clinical diagnosis and classification of the various NP syndromes for inclusion in the study were made by an experienced rheumatologist (M. Hara) and psychiatrist (K. N.), according to the standardized ACR nomenclature and case definitions for neuropsychiatric lupus syndromes [2]. These decisions were based on the medical history and neuropsychological examinations by rheumatologists, an experienced neurologist (S. U.) and a psychiatrist (K. N.) and supported by conventional laboratory tests and appropriate complementary tests, including MRI, electroencephalography, and cerebral spinal fluid tests, and the clinical course. Cases before the ACR nomenclature and case definitions for NPSLE were published in 1999 were originally diagnosed and classified according to another criteria and later re-diagnosed retrospectively according to the ACR criteria.

Clinical improvement of CNS lupus was defined as either sustained complete recovery or recovery with minor residual deficits that no longer required hospitalization. Stabilization was defined as the status in which no new clinical (i.e., neurologic or psychiatric) abnormalities occurred, although the previous abnormalities remained. Deterioration was defined as the status in which previous neuropsychiatric symptoms were exacerbated or new ones developed during follow-up [14,15].

### Evaluation of psychological impairment

Cognitive function was evaluated using the Mini Mental Status Examination [16] and the ACR-suggested test battery [2], including the WAIS-R/Digit Span (Forward) [17], Trail Making Test (Part B) [18], WAIS-R/Digit Span (Backward) [17], Wisconsin Card Sorting Test [18], Rey Auditory-Verbal Learning Test [17], WAIS-R/Block Design [17], Animal Naming Test [18], WAIS-R/Similarities [17], Trail Making Test (Part A) [18], and WAIS-R/Digit Symbol Substitution Test [17]. Mood and behavioral dysfunction was assessed by clinical observation, patient history and standardized instruments, such as the Profile of Mood States [19]. However, these formal neuropsychological tests were not performed routinely in all patients and the diagnosis of cognitive, mood, or behavioral dysfunction was on the basis of clinical assessment using the ACR definitions [2], rather than formal neuropsychological testing, especially in cases where disturbance of consciousness, such as acute confusional state, created difficulty in taking the tests. The impact of disturbance on daily life and prior occupational and social functioning was determined from information provided by the patient or other informants.

### Magnetic resonance imaging (MRI)

MRI was performed using a 1.5T MR scanner (Toshiba Medical Systems, Otawara, Tochigi, Japan) within a week from admission and within a month from onset of NP symptoms in the CNS group. Scans were aligned parallel to the axial plane through the anterior to posterior commissure and covered the entire brain in all sequences. T1-weighted, T2-weighted, and fluid-attenuated inversion recovery (FLAIR) images were acquired from all patients who had brain MRIs. The software, but not the hardware of the MR scanner was updated once in 2000, which improved its resolution but did not practically influence the sensitivity of this study. The MRIs were interpreted at the time of scanning by an experienced radiologist (T. Y.) who was not blinded to all the clinical and sequential information. MRI tests were defined as 'positive' when any abnormal intensity lesion was found. The abnormal MRI signals were classified into large abnormal MRI signals (ø ≥ 10 mm) or small abnormal MRI signals (ø < 10 mm) [15]. Mild brain atrophy, characterized by loss of brain volume, was not included among MRI abnormalities in the present study because it is not a well-established abnormality [6], even though it is the most frequent abnormal findings in SLE [9]. Moderate to severe brain atrophy was not seen at the time of enrolment in the study population. CNS lupus patients who had had abnormalities in initial MRIs were reevaluated by MRI at approximately 1 year or when clinical amelioration or deterioration was determined. MRI improvement was defined as a more than 50% decrease in the number or size of abnormal MRI signals. MRI stabilization was defined as occurring when no new abnormalities were detected, but previously detected abnormalities were unchanged or only slightly changed. Deterioration was defined as when the previously detected abnormalities became exacerbated or new ones developed during follow-up [15].

### Statistical analyses

Diagnostic tests evaluated in the present study were correlated with the final clinical diagnosis for each. Two-group comparisons were analyzed using the Mann-Whitney U test for continuous variables and Fisher's exact test for categorical variables. Values of *p *< 0.05 were considered statistically significant. Accuracy, positive predictive value (PPV), and negative predictive value (NPV) of MRI and the relative risk of CNS lupus to MRI were also calculated. All statistical analyses were performed using SPSS statistical software (version 14.0J; SPSS Inc., Tokyo, Japan).

## Results

### Clinical characteristics of the patients

Of the 191 patients with SLE enrolled in the present study, 176 were women and 15 were men. The median age of the patients was 32 years (range; 11 to 68 years). The median disease duration since the diagnosis of SLE was 1 year (range; 0 to 22 years). The patients were all Japanese, except for 1 woman who was Chinese. Current CNS syndromes were observed in 57 patients (CNS group), while the remaining 134 patients did not have either current CNS syndromes or a history of CNS syndromes (non-CNS group). Detailed clinical characteristics of the study patients are summarized in Table 1. Arthritis was significantly more frequent in the non-CNS group (*p *= 0.036) and levels of serum anti-dsDNA antibody were also significantly higher in the non-CNS group (*p *= 0.035). Although the SLEDAI score differed significantly between the 2 groups (*p *< 0.0001), the "SLEDAI score without CNS syndrome," determined by evaluating and summing the clinical variables of the SLEDAI score other than CNS syndrome for each patient, was not (Table 1).

**Table 1:** Characteristics of the 191 patients with SLE

	CNS Group	Non-CNS Group	***p***†
Characteristics	n = 57	n = 134	
			
Female/male	53/4	123/11	1.000
Age at evaluation (yrs)	28 : [23, 43]	34 : [25, 45]	0.160
SLE duration at evaluation (yrs)	1 : [0, 5]	1 : [0, 4]	0.322
Clinical features			
Malar rash/discoid rash	25 (44%)	42 (31%)	0.101
Oral or nasal ulcers	1 (2%)	13 (10%)	0.068
Arthritis	16 (28%)	60 (45%)	0.036
Serositis	7 (12%)	18 (13%)	1.000
Renal disorder	19 (33%)	37 (28%)	0.488
Vasculitis	1 (2%)	7 (5%)	0.439
Antinuclear antibody	52 (95%)	131 (98%)	0.150
Antiphospholipid antibodies‡	13 (23%)	39 (29%)	0.478
Lymphocytopenia (< 1500/mm^3^)	44 (80%)	91 (69%)	0.154
SLEDAI	15 : [10, 22]	9 : [5, 11]	< 0.0001
SLEDAI without CNS score	11 : [7, 16]	9 : [5, 11]	0.606
Anti-dsDNA antibody (RIA; IU/ml)	8 : [3, 37]	20 : [5, 98]	0.035
CH50 (U/ml)	33.2 : [21.2, 39.2]	29.8 : [20.4, 38.3]	0.496

*Except where indicated otherwise, values of continuous data are the median : [25% percentile, 75% percentile].

†*P *values were determined by Fisher's exact test or Mann-Whitney *U *test.

‡Antiphospholipid antibodies include lupus anticoagulant, anti-cardiolipin antibodies, and anti-β2GPI antibodies.

CNS: central nervous system; SLEDAI: systemic lupus erythematosus disease activity index; RIA: radioimmunoassay.

Table 2 shows the distribution of types of CNS syndrome in the study group. Neurologic disorders alone were diagnosed in 34 subjects, psychiatric disorders alone in 36, and both in 13. CNS syndromes in patients with both disorders were seizure disorders (n = 7), demyelinating syndrome (n = 4), aseptic meningitis (n = 2), acute confusional state (n = 11), anxiety disorder (n = 1) and psychosis (n = 1). The most frequent manifestation of neurologic disorders was seizures and seizure disorders (n = 18), while that of psychiatric disorders was acute confusional state (n = 24).

**Table 2:** CNS syndromes of SLE patients (n = 191)

Manifestaions of CNS lupus*	n	%	MRI positive†
Total	57	30	25 (44%)
			
Neurologic disorders	34	18	17 (50%)
Aseptic meningitis	5	3	1 (20%)
Cerebrovascular disease	1	1	1 (100%)
Demyelinating syndrome	5	3	4 (80%)
Headache‡	5	3	4 (80%)
Movement disorder (chorea)	0	0	
Myelopathy	2	1	1 (50%)
Seizures and seizure disorders	18	9	8 (44%)
			
Psychiatric disorders	36	19	16 (44%)
Acute confusional state	24	13	14 (58%)
Anxiety disorder	1	1	0 (0%)
Cognitive dysfunction§	0	0	
Mood disorders	9	5	2 (22%)
Psychosis	2	1	0 (0%)

* Based on American College of Rheumatology case definitions for neuropsychiatric ayndromes in SLE

†An MRI test was defined to be 'positive' when any abnormal intensity lesion was found.

‡Excluding tension headache (episodic tension-type headache).

§Excluding slight or mild cognitive dysfunction without significant clinical impairment as revealed by detailed neuropsychological tests.

SLE: systemic lupus erythematosus; CNS: central nervous system

### Initial MRI

All patients enrolled in the present study underwent brain MRI. We assessed the diagnostic value of brain MRI for CNS lupus using this data. Abnormal MRI signals were more frequently observed in the CNS group (n = 25, 44%) than in the non-CNS group (n = 32, 25%) with significant statistical differences [relative risk (RR), 1.7; 95% confidence interval (CI), 1.1-2.7; *p *= 0.016] (Table 3). This resulted in a diagnostic accuracy of 65%, a PPV of 42% and a NPV of 76% for MRI use in diagnosis of CNS lupus. Significant differences were also found when the CNS group with neurologic disorders (n = 17; RR, 2.3; CI, 1.3-4.1; *p *= 0.007) and the CNS group with psychiatric disorders (n = 16; RR, 1.9; CI, 1.1-3.4; *p *= 0.038) were separately compared with the non-CNS group.

**Table 3:** MRI as a diagnostic tool for CNS lupus in 191 patients with SLE

	MRI positive	MRI negative	Relative risk (95% CI)	*p*	PPV	NPV
						
CNS Group	25 (44%)	32 (56%)	1.7 (1.1 - 2.7)	0.016	0.424	0.758
Neurologic disorders	17 (50%)	17 (50%)	2.3 (1.3 - 4.1)	0.007	0.333	0.855
Psychiatric disorders	16 (44%)	20 (56%)	1.9 (1.1 - 3.4)	0.038	0.320	0.833
Non-CNS Group	34 (25%)	100 (75%)	-	-	-	-

*An MRI test was defined to be 'positive' when any abnormal intensity lesion was found.

†*P *values were determined by Fisher's exact test and CNS group or its subgroup is compared with non-CNS group.

CI: Confidence Interval; PPV: positive predictive value; NPV: negative predictive value

**Figure 1:**
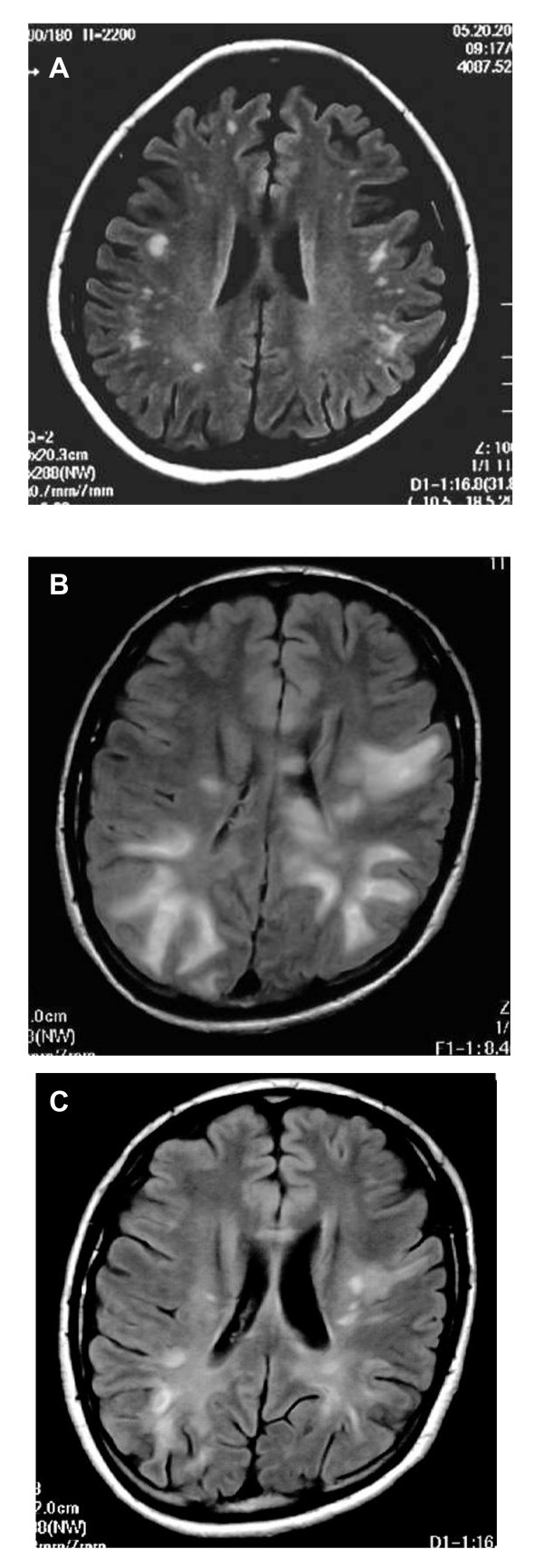
**Abnormal magnetic resonance imaging signals in patients with systemic lupus erythematosus (SLE)**. (A) Typical white-matter lesions in central nervous system (CNS) lupus in a fluid-attenuated inversion-recovery (FLAIR) image of a 21-year-old woman having a headache caused by benign intracranial hypertension. Many foci of small-sized abnormal signals are visible in the white-matter of the frontal and parietal lobes. This type of abnormality was also observed in some of the SLE patients who had no history of CNS syndrome. (B) FLAIR image of a 23-year-old woman with demyelinating syndrome and acute confusional state, showing multiple large hyperintensities involving both grey and white matter (Case 3 in Table 5). (C) Remarkable resolution of the clinical signs and imaging picture 3 months later, following high dose glucocorticoid with intravenous cyclophosphamide therapy.

Because the predictive values, in particular the PPV, of MRI were not considered adequate for the practical diagnosis of CNS lupus, we then analyzed the comparative sizes of abnormal MRI signals. Abnormal MRI signals on T2-weighted or FLAIR images of the brains of SLE patients were usually small nonspecific fixed foci of increased signal in deep white matter (Figure 1A). Mid- to large-sized high intensity lesions in brain T2-weighted or FLAIR images were occasionally observed, but only in the CNS group (Figure 1B). When abnormal signals in MRI were classified by size, large signals (ø ≥ 10 mm) were seen only in the CNS group (n = 7; 12%; RR, 3.7; CI, 2.9-4.7; *p *= 0.0002) (Table 4). Smaller signals (ø < 10 mm; mostly ø < 5 mm) were seen in both the CNS and non-CNS groups with no significant difference (n = 18; 32% and 34; 25%, respectively; RR, 1.2; CI, 0.8-2.0; *p *= 0.380) (Table 4). Calculations showed 74% accuracy, a PPV of 100% and an NPV of 73% using large abnormal MRI signals for the diagnosis of CNS lupus. Clinical syndromes of the patients showing large abnormal MRI signals included seizure disorders (n = 2), cerebrovascular disease (n = 1), demyelinating syndrome (n = 3), and acute confusional state (n = 5) (Table 5).

**Table 4:** Large MRI signal as a diagnostic tool for CNS lupus in 191 patients with SLE

	Large signal	Negative or small signal	Relative risk (95% CI)	*p*	PPV	NPV
CNS group	7 (12%)	50 (88%)	3.7 (2.9 - 4.7)	0.0002	1.000	0.728
Neurologic Disorders	5 (15%)	29 (85%)	5.6 (4.0 - 7.8)	0.0003	1.000	0.822
Psychiatric Disorders	5 (14%)	31(86%)	5.3 (3.9 - 7.3)	0.0003	1.000	0.812
Non-CNS group	0 (0%)	134 (100%)	-	-	-	-

*Abnormal MRI signals were classified into large (ø ≥ 10 mm) or small abnormal MRI signals (ø < 10 mm).

†*P *values were determined by Fisher's exact test and CNS group or its subgroup is compared with non-CNS group.

CI: Confidence Interval; PPV: positive predictive value; NPV: negative predictive value

**Table 5:** Characteristics of the CNS lupus patients with large abnormal MRI signals

Case	Duration of SLE (yrs)	SLEDAI	SLEDAI without CNS	aPL*	SLE-related CNS syndrome	Treatment	Clinical outcome of CNS lupus	MRI change
1	0	31	15	Positive	Seizure disorder, Headache	Steroids	Improve	Improve
2	0	11	3	Negative	Acute confusional state	Steroids + CPA	Improve	Improve
3	1	24	8	Negative	Demyelinating syndrome, Acute confusional state	Steroids + CPA	Improve	Improve
								
4	9	29	5	Positive	Demyelinating syndrome, Seizure disorder, Acute confusional state	Steroids + CPA	Improve	Improve
								
5	4	16	0	Negative	Demyelinating syndrome, Acute confusional state	Steroids + CPA	No change	No change
								
6	8	10	2	Negative	Cerebrovascular disease	Steroids + CPA + anticoagulant	No change	No change
								
7	5	22	6	Negative	Acute confusional state	Steroids + CPA	No change	Deteriorate

CNS: central nervous system; MRI: magnetic resonance imaging; SLE: systemic lupus erythematosus; SLEDAI: systemic lupus erythematosus disease activity index; aPL: antiphospholipid antibody; CPA: cyclophosphamide.

*aPL includes lupus anticoagulant, anti-cardiolipin antibodies, and anti-β2GPI antibodies.

†The case numbers given are not identifying numbers.

### Chronological changes in abnormal MRI signals

The association between chronological changes of abnormal MRI signals and the clinical outcome in the CNS group was examined. As shown in Tables 5 and 6, large signals always decreased in size or resolved completely in the 4 subjects where CNS syndromes were ameliorated by treatment (a representative case is shown in Figure 1B and 1C), but were unchanged in the 3 subjects where CNS syndromes did not improve; this difference was statistically significant (*p *= 0.029). Chronological changes in small signals did not correspond with clinical outcome.

**Table 6:** Large or small MRI signal as a surrogate marker for CNS lupus in 25 SLE patients

		Clinical outcome of CNS syndrome		
			
MRI signal size	MRI change	Improve	Stabilize or deteriorate	*p*
Large	Improve	4	0	0.029
	Stabilize or deteriorate	0	3	
				
Small	Improve	2	0	1.000
	Stabilize or deteriorate	13	3	

*P values were determined by Fisher's exact test.

†Large MRI signal size = ø ≥ 10 mm; Small MRI signal size = ø < 10 mm, mostly ø < 5 mm

CNS: central nervous system; MRI: magnetic resonance imaging; SLE: systemic lupus erythematosus; PPV: positive predictive value; NPV:negative predictive value

## Discussion

First, from the largest prospective cohort of SLE patients with or without active CNS syndrome, we found that abnormal signals from conventional MRI alone did not have sufficient predictive value to be practical for diagnosis of active CNS lupus, although they were associated with active CNS lupus. Second, after assortment by size, large signals (ø ≥ 10 mm) were found to occur only in the CNS group while small signals (ø < 10 mm) occurred in both the CNS and non-CNS groups with no significant difference in frequency. Finally, we did find that large signals were always reduced or resolved completely when CNS syndromes were ameliorated by treatment but did not change when CNS syndromes did not improve. In contrast, chronological changes in small signals were not related to clinical outcome. We believe this is the first report analyzing predictive values of MRI for diagnosis of active CNS lupus using a prospective study of a large cohort of patients with and without NP syndromes.

This study demonstrated that active CNS lupus was not associated with elevated "SLEDAI scores without CNS syndrome," elevated anti-DNA antibody levels, or decreased serum CH50 levels. The latter two are widely recognized and clinically used as reliable laboratory tests for estimating disease activity in patients with SLE [20,21]. Our data support the common view that CNS syndrome in SLE may occur independently from and in the absence of serological activity or other organ involvement [22].

Although we observed abnormal signal intensities in the brain MRIs of the CNS group more frequently than in those of the non-CNS group, the PPV (42%) and the NPV (76%) of MRI for detecting active CNS lupus indicate that conventional MRI alone is insufficiently specific to serve as a diagnostic tool for active CNS lupus. Active CNS lupus was diagnosed in less than half of the patients with abnormal signal intensities in brain MRI, even though normal MRI findings tended to be associated with non-CNS lupus. We also categorized patients by CNS manifestations indicating neurologic or psychiatric disorders but did not find any better results. In fact, MRI has been shown to be neither very sensitive nor specific for the diagnosis of NPSLE, estimates of sensitivity and specificity being in the 30 and 40%, respectively [12]. We would like to point out that these figures had been only estimates without solid evidence until this study. Thus, the diagnosis of CNS syndrome in SLE remains a difficult task requiring careful clinical and laboratory assessment together with such evaluation techniques as neuroimaging, electroencephalography and cerebrospinal fluid tests [7,12].

While exploring the usefulness of conventional MRI, we found that a large MRI signal is useful as a diagnostic and surrogate marker for CNS lupus. Large abnormal MRI signals (ø ≥ 10 mm) were seen only in the CNS group whereas small abnormal MRI signals (ø < 10 mm) were seen in both groups with no significant difference. In addition, chronological changes in large signals always corresponded with clinical outcome while those of small signals did not. This kind of reversible large abnormal MRI signals has been reported [4,12,15,23,24]. These large abnormal MRI signals may include new infarcts, discrete gray matter lesions, diffuse gray matter hyperintensities, and cerebral edema. These lesions, except for infarcts, are often transitory and resolve completely with time and treatment. The large abnormal MRI signals in the present study included several types of features, such as infarcts, diffuse gray matter hyperintensities, and widespread grey and white matter T2 high signal changes resembling those in acute disseminated encephalomyelitis, accompanied by various CNS syndromes. Although these large abnormal MRI signals were attributed to different pathomechanisms, they exhibited equal usefulness as a diagnostic and surrogate marker. Therefore, our simple criterion (i.e., MRI signals with ø ≥ 10 mm) is applicable to daily clinical practice without specialized knowledge of neuroimaging and regardless of the type of CNS syndromes encountered. We believe this test will prove very beneficial for clinical assessment of the condition.

In agreement with our present findings, small punctate focal lesions in the white matter have been reported to be the most common brain MRI finding, tending to be more frequently observed in the CNS than in the non-CNS SLE patients in most published reports [6,7,12,25,26]. These lesions appear anywhere in the brain, including the brain stem, and are accompanied by a variety of clinical presentation. These small punctuate hyperintense T2-weighted focal lesions are frequently nonspecific and interpreted as consistent with focal ischemia, demyelination, vasculitis, microinfarcts, gliosis, or other conditions. These small abnormal MRI signals frequently correlate poorly with clinical manifestations, and occur in patients without marked CNS signs and symptoms. In addition, conventional MRI is not capable of differentiating between older chronic and recent acute lesions of this type.

Neuroimaging must be evaluated in the context of the brain pathology of CNS lupus [4]. At this time, pathologic studies have basically been limited to post mortem studies of end stage SLE patients, which has seriously limited understanding of the sequence of events in the pathogenesis and progression of the disease [5]. Moreover, there have been few paired imaging-autopsy studies [4]. Despite the dramatic neurologic manifestations, minimal histopathologic findings in CNS lupus are typical, with apparently normal findings or nonspecific changes in the brain predominating. This general lack of specific histopathology has complicated interpretation of results found with nearly any imaging technique. For example, the autopsy of one of the patients in the present study who died from alveolar hemorrhage 5 years after showing acute confusional state and several foci of small-sized abnormal MRI signals in the white-matter showed no remarkable findings in the brain.

One of the limitations of the present study is that its subject number was insufficient for definitive conclusions about significance of conventional MRI for diagnosis of CNS lupus, although it is appreciably larger than previous studies dealing with NPSLE and MRI [15,24,27]. Another potential weakness arises from the fact that the MRI scans were not evaluated by multiple radiologists blinded to the clinical condition. Fortunately, it has been shown that evaluation of these scans is generally consistent among observers, so cranial MRI may be considered to be a valid technique to assess disease progression or reversal [15]. Because we did not include SLE patients who had CNS syndromes caused by non-SLE pathologies, the value of MRI for diagnosis of these conditions was not determined by this study. Thus, it is not clear from this study whether CNS syndromes caused by non-SLE pathologies can be differentiated by MRI, including the large MRI abnormal signals.

## Conclusions

MRI alone does not have sufficient predictive value for the diagnosis of active CNS lupus, although abnormal MRI signals showed a significant association with CNS syndrome. Importantly, a large MRI signal is a reliable and practical diagnostic and surrogate marker for CNS syndrome in SLE. The relatively small number of subjects having large MRI signals in the study indicates the need for further studies in larger numbers of patients to validate the findings.

## Competing interests

The authors declare that they have no competing interests.

## Authors' contributions

Y Katsumata conceived of the study and drafted the manuscript. M Harigai participated in data analysis and interpretation, and helped to draft the manuscript. Y Kawaguchi, CF, MS and TK participated in acquisition of data and patient recruitment. KN was responsible for psychological assessment. TY was responsible for MRI analyses. HY and M Hara participated in the design and coordination of the study. All authors read and approved the final manuscript.

## Pre-publication history

The pre-publication history for this paper can be accessed here:

http://www.biomedcentral.com/1471-2474/11/13/prepub
